# Special Issue: Character Strengths, Well-Being, and Health in Educational and Vocational Settings

**DOI:** 10.1007/s11482-018-9688-y

**Published:** 2019-01-05

**Authors:** Stefan Höfer, Fabian Gander, Thomas Höge, Willibald Ruch

**Affiliations:** 1 Medical University of Innsbruck, Innsbruck, Austria; 2 Department of Psychology, University of Zurich, Zurich, Switzerland; 3 Institute of Psychology, University of Innsbruck, Innsbruck, Austria

The evidence-based promotion of well-being for individuals, institutions and societies is a common goal amongst quality of life researchers. Central research concerns in psychology can be described as what qualities from “within” a person, or what aspects of an individuum’s personality, contribute to health and well-being? Are there stable, interindividual differences that enable and promote flourishing? And what are necessary requirements (context factors) to bring forth the qualities from “within” a person? About 15 years ago Peterson and Seligman published their seminal book “Character Strengths and Virtues. A Handbook and Classification” (Peterson and Seligman 2004). Combining cross-cultural, philosophical and theological traditions concerning the “good life” with recent psychological inquiries they developed the Values in Action (VIA) classification of strengths and virtues. They identified 24 strength presumed to contribute to the fulfillments that constitute the good life, for oneself and for others. Character strengths are personality traits that are positively valued, and represent different routes to the superordinate virtues. Short descriptions of the character strengths are provided in Table 1.

To be included in the VIA classification and considered a character strength, a potential strength had to fulfill a minimum set out of ten criteria, including being morally valued in its own rights; being traitlike, manifest in thoughts, feelings, and behavior, and somewhat consistent across situations and time; and being distinct from the other character strengths in the classification (Peterson and Seligman 2004).

Thereby the authors reclaimed the study of character and virtue as legitimate topics of psychological research that has been neglected for a long time, since psychology focused on studying value-neutral personality traits (see for example Allport 1921). However, when we are interested in studying positive outcomes, in particular health and well-being, positive valued characteristics – such as character strengths – can be expected to be much more relevant than presumably neutral concepts.

This international special issue of *Applied Research in Quality of Life* focuses on the broad area of character strengths, well-being, and health research, also including (but not limited to) research and applications of well-being and positive psychological concepts in educational and vocational settings. The studies reported in this special issue are of high quality and diverse methodology. They span from cross-sectional studies based on large panel datasets (more than 15,000 participants) over longitudinal studies with more specific samples to the development of theory and application. All papers followed the standard peer-review process of the journal; papers authored or co-authored by the editors were assigned to action editors, who independently carried out the review process.

The first set of papers by Wagner et al. (2018), Baumann et al. (2018), Gander et al. (2018) and Hofmann et al. (2018) address the issues how well-being and character strengths from a basic scientific point of view are interlinked. Against the background of the PERMA-model by Seligman (2011) suggesting five dimensions of flourishing and well-being that encompass both subjective and eudemonic aspects, Wagner et al. (2018) demonstrated in a sample of over 5000 participants the robust relationships of character strengths with these different dimensions of well-being. Further, these relationships also hold when not relying only on self-ratings but also taking informantratings into account. Baumann et al. (2018) focus on life satisfaction in later life, in particular how several transitions specific for older people (e.g., retirement or being widowed) are linked to life satisfaction and character strengths. Based on data of over 15,000 older adults the authors conclude that character strengths might offer a starting point for strengths-based intervention programs to maintain, enhance, or prevent a decrease in life satisfaction in later life. Despite character strengths being seen as stable traits, they are also considered malleable due to cultivation or deliberate interventions. Accordingly, Gander et al. (2018) explored the stability and malleability of character strengths and their relations to well-being applying a longitudinal study with more than 1700 participants. Their results provide an important basis for intervention studies addressing the promotion of character strengths and well-being of individuals. The paper of Hofmann et al. (2018) focuses on one specific aspect of a virtuous character, *humor,* and examines its relationships to another desirable personal characteristic, mindfulness. The authors report three empirical studies in different settings including nearly 600 participants in total. Their core hypotheses proposed that mindfulness is positively linked to “light” forms of humor and negatively linked to darker forms. Accordingly, Study 1 and Study 2 investigated the relationship between different aspects of humor and mindfulness. Additionally, in Study 3 the effects of a mindfulness intervention on humor were examined concluding that it might be fruitful to combine humor interventions and mindfulness interventions.

Udayar and colleagues (Udayar et al. 2018) broadened the scope by investigating character strengths not directly, but focusing on the development of the basic “big five” dimensions of personality. Based on a cross-lagged-panel design over 4 years and data from more than 1300 individuals Udayar et al. (2018) demonstrate that perceived social support has positive effects on the development of these “big five” personality traits in middle adulthood. Because previous research showed that big five personality traits are related to character strengths their results hint towards not only the malleability of fundamental psychological traits but also character strengths by social support.

Well-being at work and in occupational settings is an important topic in real life what is well-reflected by its increased coverage in research, but also the mainstream media (Danna and Griffin 1999). Research on character strengths might allow for broadening the perspective and providing insights about what can go “right” at the workplace, thereby not only preventing negative consequences, but also fostering positive outcomes, such as well-being, engagement, or a positive organizational climate. Thus, understanding factors contributing to well-being and health in the work and organizational setting considering the role of character strengths is a promising field for both research and application. Based on a sample of hospital physicians the paper by Huber et al. (2018) addresses the question whether the *possession* of certain character strengths is enough to enhance well-being at work or if the *possibility to apply* one’s character strengths at work is mandatory to enhance well-being. Based on cross-sectional and longitudinal data also from the field of hospital physicians the studies by Strecker et al. (2018) and Höge et al. (2018), identified condition-related *antecedents* of the applicability of individual signature character strengths at work and their relation to work engagement and well-being. Strecker et al. (2018) focused on task related work characteristics (e.g., autonomy, social support, learning demands) whereas Höge et al. (2018) addressed a specific subset of the organizational climate, the so-called “socio-moral climate” as possible antecedent. Also addressing condition-related effects on strength use at work and its positive outcomes, Meyers et al. (2018) demonstrate that organizational support for strength use at work is positively related to work engagement and contextual performance, and that this association is stronger among younger employees. Heintz and Ruch (2018) focus on a different fulfillment in life, namely the satisfaction derived from with one’s work. They investigate the relationship between the 24 character strengths (and five strengths factors) with components of job satisfaction and overall job satisfaction in a general working population (*N* = 12,499) to identify the list of strengths that facilitate being satisfied with work in general. The authors argue that knowing which strengths are more important for specific working populations can help to develop and apply more effective strength-based interventions in the workplace, thereby improving positive and reducing negative work-related outcomes.

Littman-Ovadia and Freidlin (2018) focus in their work on the overuse and underuse of character strengths and which implications this may have towards flourishing, satisfaction with life, and psychopathology such as obsessive-compulsive disorder. In relation to this, Niemiec (2018) provides theoretical thoughts on six different functions of character strengths for flourishing in times of adversity and opportunity. These six functions provide a promising new framework for future research and practice. In her literature review Lavy (2018) critically addresses the potential of teaching character strengths at schools. In particular, she identifies obstacles to integrate such activities in the school curricula. However, she also adds a strong note of caution to the notion that character strengths are only and ultimately positive.

Finally, the methodology of measuring character strengths is essential to have reliable and valid data. The paper of Höfer et al. (2018) reports the psychometric properties of a German character strengths short form measure based on data from two independent samples.

This special issue critically highlights the concept of character strengths in relation to well-being and provides new scientific evidence and a conceptual theoretical framework to further develop quality of life research from the perspective of Positive Psychology.

## Figures and Tables

**Table 1:** The VIA classification of character strengths and their assignment to the six virtues

**1. Wisdom and knowledge – cognitive strengths that entail the acquisition and use of knowledge**
*Creativity [originality, ingenuity]*: Thinking of novel and productive ways to conceptualize and do things; includes artistic achievement but is not limited to it
*Curiosity [interest, novelty-seeking, openness to experience]*: Taking an interest in all of ongoing experience for its own sake; finding subjects and topics fascinating; exploring and discovering
*Judgment [open-mindedness, critical thinking]*: Thinking things through and examining them from all sides; not jumping to conclusions; being able to change one’s mind in light of evidence; weighing all evidence fairly
*Love of Learning*: Mastering new skills, topics, and bodies of knowledge, whether on one’s own or formally; obviously related to the strength of curiosity but goes beyond it to describe the tendency to add systematically to what one knows
*Perspective [wisdom]*: Being able to provide wise counsel to others; having ways of looking at the world that make sense to oneself and to other people
**2. Courage – emotional strengths that involve the exercise of will to accomplish goals in the face of opposition, external or internal**
*Bravery [valor]*: Not shrinking from threat, challenge, difficulty, or pain; speaking up for what is right even if there is opposition; acting on convictions even if unpopular; includes physical bravery but is not limited to it
*Perseverance [persistence, industriousness]*: Finishing what one starts; persisting in a course of action in spite of obstacles; “getting it out the door”; taking pleasure in completing tasks
*Honesty [authenticity, integrity]*: Speaking the truth but more broadly and presenting oneself in a genuine way and acting in a sincere way; being without pretense; taking responsibility for one’s feelings and actions
*Zest [vitality, enthusiasm, vigor, energy]*: Approaching life with excitement and energy; not doing things halfway or halfheartedly; living life as an adventure; feeling alive and activated
**3. Humanity – interpersonal strengths that involve “tending and befriending” others**
*Love [capacity to love and be loved]*: Valuing close relations with others, in particular those in which sharing and caring are reciprocated; being close to people
*Kindness [generosity, nurturance, care, compassion, altruistic love,* “*niceness*”*]*: Doing favors and good deeds for others; helping them; taking care of them
*Social Intelligence [emotional intelligence, personal intelligence]*: Being aware of the motives and feelings of other people and oneself; knowing what to do to fit into different social situations; knowing what makes other people tick
**4. Justice – civic strengths that underlie healthy community life – strengths of justice**
*Teamwork [citizenship, social responsibility, loyalty]*: Working well as a member of a group or team; being loyal to the group; doing one’s share
*Fairness*: Treating all people the same according to notions of fairness and justice; not letting personal feelings bias decisions about others; giving everyone a fair chance
*Leadership*: Encouraging a group of which one is a member to get things done and at the time maintain time good relations within the group; organizing group activities and seeing that they happen
**5. Strengths that protect against excess – strengths of temperance**
*Forgiveness [mercy]*: Forgiving those who have done wrong; accepting the shortcomings of others; giving people a second chance; not being vengeful
*Humility [modesty]*: Letting one’s accomplishments speak for themselves; not regarding oneself as more special than one is
*Prudence*: Being careful about one’s choices; not taking undue risks; not saying or doing things that might later be regretted
*Self-Regulation [self-control]*: Regulating what one feels and does; being disciplined; controlling one’s appetites and emotions
**6. Transcendence – strengths that forge connections to the larger universe and provide meaning**
*Appreciation of Beauty and Excellence [awe, wonder, elevation]*: Noticing and appreciating beauty, excellence, and/or skilled performance in various domains of life, from nature to art to mathematics to science to everyday experience
Gratitude: Being aware of and thankful for the good things that happen; taking time to express thanks
*Hope [optimism, future-mindedness, future orientation]*: Expecting the best in the future and working to achieve it; believing that a good future is something that can be brought about
*Humor [playfulness]*: Liking to laugh and tease; bringing smiles to other people; seeing the light side; making (not necessarily telling) jokes
*Spirituality [religiousness, faith, purpose]*: Having coherent beliefs about the higher purpose and meaning of the universe; knowing where one fits within the larger scheme; having beliefs about the meaning of life that shape conduct and provide comfort

Adapted from VIA Institute on Character, © Copyright 2004–2018. All Rights Reserved. Used with Permission. www.viacharacter.org
